# Comparison of perioperative outcomes of open (CUSA) versus laparoscopic (LOTUS) major hepatectomy – revisited. First evaluation of efficacy and safety of AEON™ stapler

**DOI:** 10.3389/fonc.2025.1616876

**Published:** 2025-07-23

**Authors:** Affan Iqbal, Minas Baltatzis, Panagiotis Stathakis, Jenifer Barrie, Ajith Kumar Siriwardena, Saurabh Jamdar, Aali Jan Sheen

**Affiliations:** ^1^ Dept of Hepatopancreatobiliary (HPB), Manchester Foundation National Health Service (NHS) Trust, Manchester, United Kingdom; ^2^ Dept of General Surgery, Northern Care Alliance Foundation National Health Service (NHS) Trust, Manchester, United Kingdom; ^3^ Faculty of Biology, Medicine and Health, University of Manchester, Manchester, United Kingdom

**Keywords:** LOTUS liver energy, laparoscopic liver resection, AEON endovascular stapler, major hepatectomy, propensity score matching

## Abstract

**Introduction:**

In 2019, preliminary data matching 20 laparoscopic (using LOTUS) with 20 open (using CUSA) cases demonstrated the feasibility and improved outcomes of laparoscopic major liver resections using the LOTUS™ liver blade. This updated study presents a larger comparison between open and laparoscopic major hepatectomies and, for the first time, evaluates the safety of the endovascular stapler AEON™ (Lexington Medical, Massachusetts, USA).

**Methods:**

All consecutive patients who underwent liver resections, both open and laparoscopic, from January 2020 to June 2023 were identified from a prospectively maintained database. Propensity score matching was performed to identify matched open and laparoscopic cases, which were compared for intra- and post-operative short-term outcomes. The LOTUS™ ultrasonic energy device was used for transection in laparoscopic cases, whereas CUSA was used in open procedures. AEON was introduced in 2021 and compared with the previously used stapler.

**Results:**

The initial sample of 116 patients was narrowed to 86 after applying 1:1 matching. The median age was 63 years (range 27–83). Laparoscopic cases showed reduced hospital stay (mean 7.8 vs. 14.7 days; p = 0.025), increased Pringle time (48.5 vs. 33 minutes; p = 0.010), and reduced transfusion requirements (0 vs. 4 units; p = 0.035). Comparing AEON™ with Endo-GIA showed no statistical differences, though AEON™ showed a possible trend toward reduced transection time overall (56 vs. 69 minutes; p = 0.300) and in laparoscopic cases (56 vs. 71 minutes; p = 0.295).

**Conclusion:**

The LOTUS™ liver blade continues to demonstrate safety and efficacy in laparoscopic liver resections. Transection time has improved compared to the earlier study, likely reflecting increased experience. AEON™ is shown to be non-inferior for vessel ligation, with a potential trend toward reduced transection time in both open and laparoscopic cases.

## 1 Introduction

In 2019, our unit published its initial experience with laparoscopic major liver surgery using the LOTUS ultrasonic energy device (BOWA-electronic GmbH, Gomaringen, Germany), demonstrating the feasibility and promising clinical outcomes of this approach in comparison to conventional open surgery using CUSA (Cavitron Ultrasonic Surgical Aspirator) (1). Over the past two decades, laparoscopic liver resection has increasingly become associated with improved perioperative outcomes, notably reduced hospital stay and analgesic requirements, without compromising safety or oncological efficacy (2, 3). Although early randomised controlled trials suggested equivalence between laparoscopic and open approaches, limitations in trial design—such as early termination—have restricted their strength of evidence (4). However, meta-analyses have shown significant advantages for the laparoscopic approach, particularly in left lateral sectionectomy, where reduced length of stay, lower transfusion and reoperation rates, and improved overall outcomes have been observed (5). Economic comparison between AEON and Endo-GIA staplers was intended but could not be completed due to incomplete data. Specifically, itemised cost data for the devices and associated consumables were unavailable for a significant proportion of cases, precluding a meaningful cost-effectiveness analysis. This limitation reflects current institutional data recording practices and should be addressed in future studies.

Based on international consensus (Morioka, Japan, 2015) (6), laparoscopic major hepatectomy is defined to include hemihepatectomies, trisectionectomies, and resections of the posterior superior segments (IVa, VII, VIII)—procedures that remain technically challenging within minimally invasive surgery (1). Recent data further support laparoscopic major resections, including posterior segmentectomy, suggesting that with increasing experience, comparable or improved outcomes may be achieved (7, 8).

No singular transection technique has yet been universally adopted as superior in laparoscopic liver surgery (9). However, the LOTUS liver blade has demonstrated a particular advantage due to its ultrasonic cutting capability with simultaneous haemostasis of vessels up to 4 mm in diameter (1). This updated appraisal evaluates a more extensive dataset of 116 patients over a three-year period (2020–2023), with 43 matched pairs following propensity score matching. Importantly, this study examines how operative outcomes continue to improve with experience, particularly in relation to transection time using the LOTUS blade, underscoring a learning curve and refinement of technique.

Furthermore, this is the first published evaluation of the AEON™ endovascular stapler (Lexington Medical, Boston, USA) in liver surgery, utilising advanced S3 engineering tristaple technology. While AEON is newly introduced to hepatobiliary practice, this study appraises its safety and efficacy in comparison to the widely used Endo-GIA stapler. Although no statistically significant differences were found in perioperative outcomes, a trend towards reduced transection time was noted, supporting the hypothesis that with increasing operator familiarity, AEON’s role in hepatic parenchymal and vascular division may further evolve and improve. This paper therefore provides a critical appraisal of novel tools in laparoscopic liver surgery and how their performance is expected to develop with growing clinical experience.

## 2 Patients and methods

This is an updated single centre clinical cohort study based on a retrospective analysis of prospectively collected data on patients who underwent major liver resection by either the open or laparoscopic routes in the tertiary regional hepato-pancreatobiliary (HPB) centre of Manchester Royal Infirmary during the period January 2020 to January 2023. It is based on a consecutive series of patients operated by two consultant hepatobiliary surgeons and represents an update of the previously reported data in 2019. Patients were identified from databases maintained prospectively by the unit data manager. All major resections carried out were included. Open resections for perihilar cholangiocarcinomas were excluded, as there were no comparators in the laparoscopic group. Propensity score matching analysis was performed in the original sample, which resulted in a smaller number of matched open and laparoscopic cases, which were used for comparison. Both open and laparoscopic techniques used as well as the preoperative assessment were as previously described (1). Blood loss was taken from the volume in the suction bottles at the end of each procedure, swabs though were not routinely weighed.

### 2.1 Data collection

Data from the two surgeons’ (AS & SJ) were collected from the unit’s data manager and were split into 4 main categories: (a) demographic details (age, gender, World Health Organization performance status score), (b) disease related parameters (histological diagnosis, unilobar/bilobar liver disease, neoadjuvant chemotherapy details), (c) surgical procedure details (type of resection, duration of surgery, duration of Pringle manoeuvre, parenchymal transection time, transfusion rate), and outcome (resection margin status, postoperative morbidity using Clavien-Dindo classification, hospital stay, 30-day readmission and mortality).

### 2.2 Data availability

The datasets used and/or analysed during the current study available from the corresponding author on reasonable request.

### 2.3 Ethics

The study was categorized as an audit by the Manchester University Hospitals Foundation Trust Research and Development office and was registered with the hospital’s audit department, so consent from patients specifically was not required. The study was conducted in accordance with institutional ethical guidelines and the principles of the Declaration of Helsinki. Given the retrospective nature of this analysis, formal patient consent was waived, as all data were anonymised prior to analysis. In addition, ethics committee approval was sought with NHS health authority and regarded as not required as per a decision made by a trust research committee after using the NHS Health Research Authority (HRA) decision toolkit https://www.hra-decisiontools.org.uk/research/.

### 2.4 Statistical analysis

**Figure 1:**
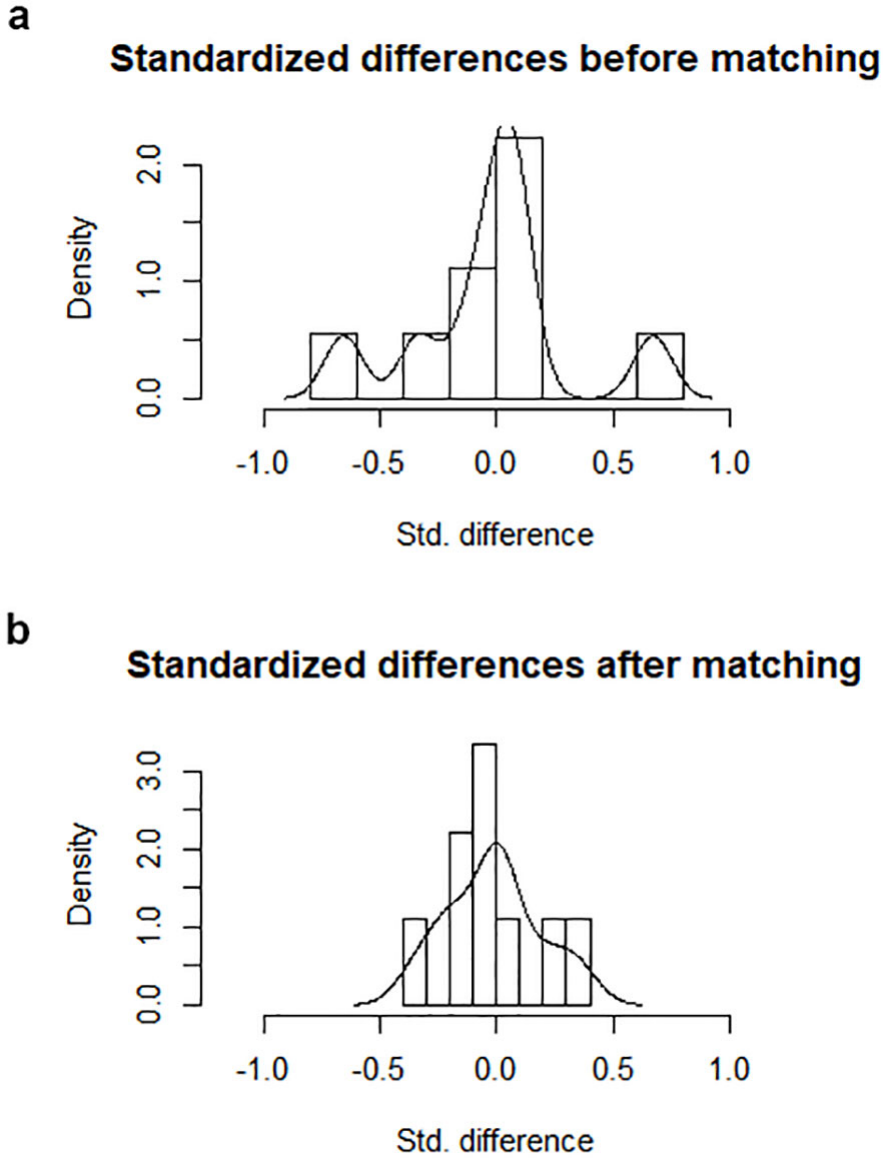
**(a)** Standardized differences within the sample before and after the matching process. **(b)** Absolute standardized difference in the matched data compared with the original data.

Propensity score matching diagnostics were evaluated using standardised mean differences before and after matching. **Figure 1a** demonstrates the reduction in standardised differences post-matching, confirming adequate balance across covariates. **Figure 1b** further illustrates the improved balance with lower absolute standardised differences, ensuring the comparability of the matched cohorts. A brief summary of these diagnostics has been included to support the robustness of the matching process for readers less familiar with the methodology.

Propensity score matching analysis was performed using the R statistical tool for SPSS (IBM Corp; IBM SPSS Statistics for Windows, Version 23.0, Armonk, NY, USA). Nearest neighbour was the matching algorithm used in our analysis, with a match ratio of 1:1. Caliper value was set to 0.2. Patients’ characteristics selected for the matching analysis were the following: age, administration of chemotherapy prior to resection, colorectal liver metastases vs. other malignancies, disease distribution (unilobar vs. bilobar) and WHO performance status score. The number of matching criteria is inversely proportional to the size of the post-matching sample, so the more the matching criteria, then more cases are discarded by the statistical software. Taking this into consideration, only five matching criteria were used, aiming to maintain the final cohort size and the statistical power of the study at acceptable levels. Outcome comparisons were performed using One-Way ANOVA and Chi-Square test in SPSS. Statistical significance was defined as p < 0.05.

### 2.5 AEON endovascular stapler

The AEON surgical stapler, like other available surgical staplers, is used to simultaneously cut and staple tissue when resecting, transecting, or creating anastomoses. The product is used across surgical specialties, including general, abdominal, gynaecologic, paediatric, thoracic, bariatric, colorectal, urological, and of course HPB surgery. The instrument can be utilised in both open and minimally invasive laparoscopic surgery. It was introduced in the unit mainly as a result of its subjective improved handling properties with increased articulation and a smoother firing ratchet mechanism. It employs what is described as S3 engineering technology, which has shown in the unit to decrease pancreatic fistula rates in distal pancreatectomy procedures (10). The first technical attribute of S3 Engineering is the AEON superior staple line. AEON is engineered to deploy a staple line that minimizes bleeding and leaks. This is achieved by maximizing the number of properly formed staples in each firing, by maximizing the strength of the staple line, by ensuring a clean-cut line, and by deploying three staggered rows of staples with uniform staple height.

## 3 Results

### 3.1 Study characteristics

From January 2020 to June 2023–116 consecutive patients underwent major hepatectomy under the care of two HPB consultants. 73 of them (63%) had open major hepatectomy and 43 (37%) laparoscopic. This represents an increase of the proportion of laparoscopic cases compared to the previous study by 11%. The indications for surgery were metastases from colorectal cancer in 81 patients (70%) and other malignant tumours in the remaining 35 patients (30%). Right or left hemihepatectomy was performed in the majority of cases (79%), trisectionectomies in 5% and laparoscopic major resections including posterior segments (4a, 7,8), as per Louisville consensus definition (1), in the remaining 16%.

### 3.2 Propensity score matching analysis

Propensity score matching analysis was performed based on the aforementioned matching criteria and resulted in 43 patients in each group and 86 in total (20 in each group on the previous study). The matched subgroup of patients was used for the outcome comparisons. The results of the matching analysis are summarised on **Table 1**. **Figure 1a** demonstrates the decreased standardized differences within the sample after the matching process. **Figure 1b** shows lower absolute standardized difference in the matched data compared with the original data. The matching criteria (age, WHO performance status score, disease diagnosis and distribution, administration of chemotherapy prior to resection) did not differ significantly between the open and laparoscopic groups within the matched sample. The median age of the entire cohort of the matched 86 patients was 63 years of age (range 27-83). Male and female patients were almost equally represented in both groups (p=0.864).

**Table 1:** Propensity score matching analysis - summary.

Subsamples	All		Matched		Unmatched		Discarded	
	Open	Laparoscopic	Open	Laparoscopic	Open	Laparoscopic	Open	Laparoscopic
N	73	43	43	43	30	0	0	0
Total	116		86		30		0	

### 3.3 Short term outcome comparison

Similarly to the previous study the mean duration of laparoscopic cases was slightly longer (266 versus 238min), but this finding was not statistically significant (p=0.189). Pringle manoeuvre was as previously described, used for all cases and was significantly longer in laparoscopic compared to open surgery 48 vs 33min, p=0.01). The duration of Pringle was, however shorter than in the previous study for both the entire cohort and the 2 groups. In terms of achieving complete tumour resection the R0 rates were comparable in both groups (p=0.201). The duration of parenchymal transection, measured longer in laparoscopic hepatectomies in the original study, was found similar in the newer cohort (p=0.935).

**Figure 2:**
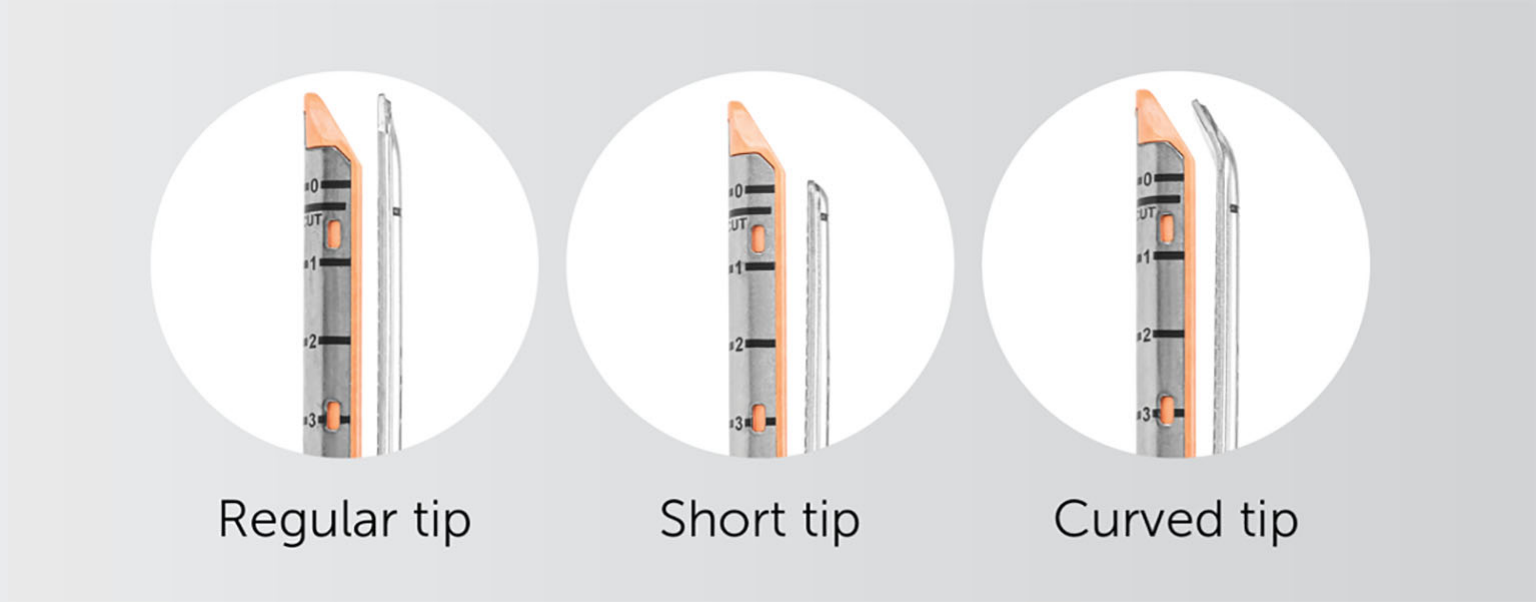
– AEON endovascular stapler has an innovative design with three tips available to the surgeon, with the curved tip excellent in isolating major vascular structures (used with permission for publication by Lexington medical, Boston, USA).

Requirements for intraoperative transfusion were similar in both groups (p=0.306). On the contrary 4 patients required postoperative transfusion after open surgery versus 0 in the laparoscopic group, which was statistically significant. As depicted in the earlier report in 2019, the benefit of reduced hospital stay after laparoscopic surgery was confirmed (8 vs 15 days after open hepatectomies, p=0.025). Similar readmission rates were observed in both groups (p=0.152). The above findings are summarised in **Table 2** and **Figures 1a, b**, **2**.

**Table 2:** Comparison of the intraoperative and postoperative outcome between the open and the laparoscopic groups.

	Open (n=73)	Laparoscopic (n=43)	P value
**Duration of surgery** (min, mean ± SD)	238.2 ± 98.4	266 ± 94.9	0.189
**Duration of Pringle Maneuveur** (min, mean ± SD)	32.8 ± 24.5	48.5 ± 27.8	**0.010**
**Parenchymal transection time** (min, mean ± SD)	67.3 ± 33.2	66.6 ± 36.1	0.935
Transfusion			
Intraoperative (n,%)^†^	8/41 (19%)	2/38 (5%)	0.087
Postoperative (n,%) ^†^	4/37 (11%)	0/39 (0%)	**0.035**
Complications			
Clavien-Dindo I+II (n,%)	5/43 (12%)	8/43 (19%)	0.391
Clavien-Dindo III+IV (n,%)	9/43 (21%)	4/43 (9%)	0.132
Total (n,%)	14/43 (33%)	12/43 (28%)	0.407
**Mortality (n,%)**	0	0	1.000
**R0 resection margin (n,%)**	28/43 (65%)	34/43 (79%)	0.201
**Hospital stay** (days, mean ± SD)	14.67 ± 18.91	7.8 ± 5.75	**0.025**
**30-day readmission (n,%)**	0/43 (0%)	2/43 (5%)	0.152

^†^Missing data.

On subset analysis, 17 laparoscopic major hepatectomies were performed using the AEON vascular stapler between May 2022 and January 2023. There was no difference in any of the outcomes when comparing these cases with the remaining cohort. It is worth noting that the intraoperative and postoperative transfusion rate was zero with AEON versus 15% and 7% respectively in the cases performed with other vascular stapler devices, although this finding was not statistically significant. Outcome comparison was also performed between AEON and other staplers among the laparoscopic cases only. Once more AEON stapler was not inferior in any of the outcomes measured (**Tables 3**, **4**)(please also see **Appendix Figures 3**, **4**).

**Table 3:** Comparison of the intraoperative and postoperative outcome between AEON and other staplers.

	AEON	Other staplers	P value
**Duration of surgery** (min, mean ± SD)	272.2 ± 65.5	247.6 ± 102.7	0.379
**Duration of Pringle Maneuveur** (min, mean ± SD)	45.3 ± 22	39 ± 27.9	0.484
**Parenchymal transection time** (min, mean ± SD)	55.9 ± 20.4	68.7 ± 35.7	0.300
Transfusion			
Intraoperative (n,%) ^†^	0/14 (0%)	10/65 (15%)	0.102
Postoperative (n,%) ^†^	0/15 (0%)	4/61 (7%)	0.308
Complications			
Clavien-Dindo I+II (n,%)	4/17 (24%)	9/69 (13%)	0.292
Clavien-Dindo III+IV (n,%)	1/17 (6%)	12/69 (17%)	0.235
Total (n,%)	5/17 (29%)	21/69 (30%)	0.934
**Mortality (n,%)**	0	0	1.000
**R0 resection margin (n,%)**	15/17 (88%)	47/69 (68%)	0.114
**Hospital stay** (days, mean ± SD)	7.4 ± 5.6	12.2 ± 15.6	0.220
**30-day readmission (n,%)**	0/17 (0%)	2/69 (3%)	0.478

^†^Missing data.

**Table 4:** Comparison of the intraoperative and postoperative outcome between AEON and other staplers within laparoscopic cases.

	AEON	Other staplers	P value
**Duration of surgery** (min, mean ± SD)	272.2 ± 65.5	262,5.6 ± 109.4	0.757
**Duration of Pringle Manoeuvre** (min, mean ± SD)	45.3 ± 22	50 ± 30.4	0.647
**Parenchymal transection time** (min, mean ± SD)	55.9 ± 20.4	71.2 ± 40.6	0.295
Transfusion			
Intraoperative (n,%) ^†^	0/14 (0%)	2/22 (9%)	0.540
Postoperative (n,%) ^†^	0/15 (0%)	0/26 (0%)	1.000
Complications			
Clavien-Dindo I+II (n,%)	4/17 (24%)	4/26 (18%)	0.502
Clavien-Dindo III+IV (n,%)	1/17 (6%)	3/26 (12%)	0.532
Total (n,%)	5/17 (29%)	7/26 (27%)	0.859
**Mortality (n,%)**	0	0	1.000
**R0 resection margin (n,%)**	15/17 (88%)	19/26 (73%)	0.098
**Hospital stay** (days, mean ± SD)	7.4 ± 5.6	8.1 ± 5.9	0.716
**30-day readmission (n,%)**	0/17 (0%)	2/24 (8%)	0.242

^†^Missing data.

*Summary in comparison to earlier report;* Comparing open vs laparoscopic approaches, hospital stay as previously reported was shorter for laparoscopic cases (P=0.025), duration of surgery remained longer but not significantly (P=0.123). In addition, transection time was quicker for laparoscopic surgery, which may suggest an improvement after a learning curve was achieved in using the LOTUS device (P=0.935), but the pringle time remained significantly longer (P=0.005) (this data is not tabulated in the results).

## 4 Discussion

Laparoscopic liver resection (LLR) continues to gain traction globally, driven by improvements in surgical technique, equipment, and operator experience. Since our unit’s original report in 2019 evaluating the feasibility of major laparoscopic hepatectomy using the LOTUS™ ultrasonic liver blade (1), the technique has matured, and its integration into complex resections has broadened. Numerous centres have reported similar findings, showing that LLR achieves acceptable morbidity, shorter hospital stays, reduced transfusion rates, and equivalent oncological outcomes when compared with open surgery (2, 11, 12). Randomised controlled trials have further validated these benefits, despite challenges in accrual and heterogeneity of procedures (4, 13–15).

The OSLO-COMET trial demonstrated that LLR significantly reduces length of stay (53 vs. 96 hours, P < 0.001) and achieves cost neutrality, challenging concerns about the financial implications of newer technologies (7). Subgroup analysis of resections involving posterior segments also reported shorter hospital stays in the laparoscopic group (2 vs. 4 days, P < 0.001) (8). Our study adds to this growing body of literature and places a specific focus on technological evolution—particularly with the LOTUS™ liver blade—and how its increased use has translated into measurable improvements in operative performance.

Our updated cohort revealed that transection time, initially longer in laparoscopic cases in the 2019 series (1), is now comparable to open surgery (P = 0.935), reflecting both institutional experience and technical refinement. Importantly, our data also show a significant reduction in transfusion requirements (0 vs. 4 units, P = 0.035) and length of hospital stay (7.8 vs. 14.7 days, P = 0.025) in laparoscopic cases. These improvements align with wider trends in the literature and demonstrate that the LOTUS™ blade is not only safe and effective, but also increasingly efficient with experience.

The LOTUS™ device allows for simultaneous transection and vessel sealing, eliminating the need for multiple instruments typically required with CUSA, such as clip applicators and separate coagulation devices. This simplification is particularly advantageous in the confined working space of laparoscopic surgery, reducing time-consuming instrument exchanges and contributing to smoother operative flow. The rise in laparoscopic major hepatectomies at our centre—from 26% to 37%—further reflects growing confidence in the approach and the reliability of the device.

In this series, cumulative Pringle time remained longer in laparoscopic procedures compared to open. This may reflect the careful, staged approach often adopted in laparoscopic cases, where immediate control of bleeding can be more technically challenging. However, pneumoperitoneum has been shown to reduce blood loss by increasing intra-abdominal pressure, which may help offset this challenge during liver transection (16).

While this study also introduced the AEON™ endovascular stapler to the liver surgery setting, it played a complementary role. AEON™ demonstrated non-inferiority to the Endo-GIA™ stapler across all matched outcomes, with a trend toward reduced transection time (56 vs. 69 minutes, P = 0.300 overall; 56 vs. 71 minutes, P = 0.295 laparoscopic cases). Although not statistically significant, these findings suggest that AEON™ may have a role in improving efficiency as surgeons gain experience with its ergonomics and cartridge design. Features such as the curved tip and shorter profile are particularly suited for intraparenchymal dissection and precise vascular control. Although our subgroup analysis of AEON stapler use suggests a possible trend toward improved outcomes, the small sample size (n=17) is underpowered for drawing definitive conclusions. Non-significant p-values should not be interpreted as meaningful trends in isolation and should ideally be supported by confidence intervals or larger sample validation.

Nevertheless, the main advancement illustrated in this study is attributable to the LOTUS™ energy device. It offers significant procedural advantages by simplifying laparoscopic parenchymal transection, enabling precise dissection while reducing bleeding risk and overall operative time. These findings are consistent with the ORANGE II randomised trial, which supports the feasibility and safety of laparoscopic major liver surgery (8, 17).

It is worth noting that CUSA, although widely used in open liver surgery, has less practicality in laparoscopy due to the number of required instruments and the need for coordinated use by multiple hands. In contrast, the LOTUS™ device offers a more adaptable two-instrument setup (LOTUS + stapler), making it better suited to the laparoscopic environment. This shift may also yield efficiency and cost advantages that merit further evaluation. As various tools for parenchymal transection continue to evolve, future comparisons of ultrasonic devices, CUSA, and robotic platforms will be essential in determining the most effective and economical options in minimally invasive liver surgery (18).

## 5 Limitations

This study has several limitations:

### 5.1 Retrospective design

Although propensity score matching was used, selection bias cannot be fully excluded.

### 5.2 Single-centre experience

Limits generalisability to other surgical settings and institutions.

### 5.3 Small AEON™ cohort

The sample size for AEON™ subgroup analysis is limited and underpowered.

### 5.4 Short follow-up

Long-term oncological outcomes and late complications were not assessed.

### 5.5 Lack of cost data and cartridge count

Economic analysis was not possible due to incomplete data.

Future prospective, multi-centre studies should assess these variables more comprehensively and explore comparative analysis between energy platforms in laparoscopic liver surgery.

## 6 Conclusion

This updated analysis demonstrates that the LOTUS™ ultrasonic liver blade remains a safe, effective, and increasingly efficient tool for laparoscopic major hepatectomy. Improvements in transection time, blood conservation, and hospital discharge reflect both the performance of the device and the learning curve achieved by surgeons using it. While the AEON™ stapler shows non-inferior results and has potential utility, particularly in ergonomic handling and vascular control, the core innovation driving progress in this study remains the LOTUS™ device. Continued research is needed to confirm these findings in larger, diverse populations and to refine the role of these technologies in the broader context of minimally invasive liver surgery. While this study offers valuable single-centre insights into the use of AEON and LOTUS devices in major hepatectomy, randomised controlled trials and multi-centre studies will be essential to validate these findings and establish broader applicability.

## Data Availability

The raw data supporting the conclusions of this article will be made available by the authors, without undue reservation.
