# Supplementary material for: Comparison of perioperative outcomes of open (CUSA) versus laparoscopic (LOTUS) major hepatectomy – revisited. First evaluation of efficacy and safety of AEON™ stapler
Source: Front Oncol. 2025 Jul 23;15:1616876. doi: 10.3389/fonc.2025.1616876 (PMC12325070; doi:10.3389/fonc.2025.1616876)

Appendix

Figure 2: Graph comparing the mean duration of surgery (with 95% Cl) between open and laparoscopic major hepatectomies


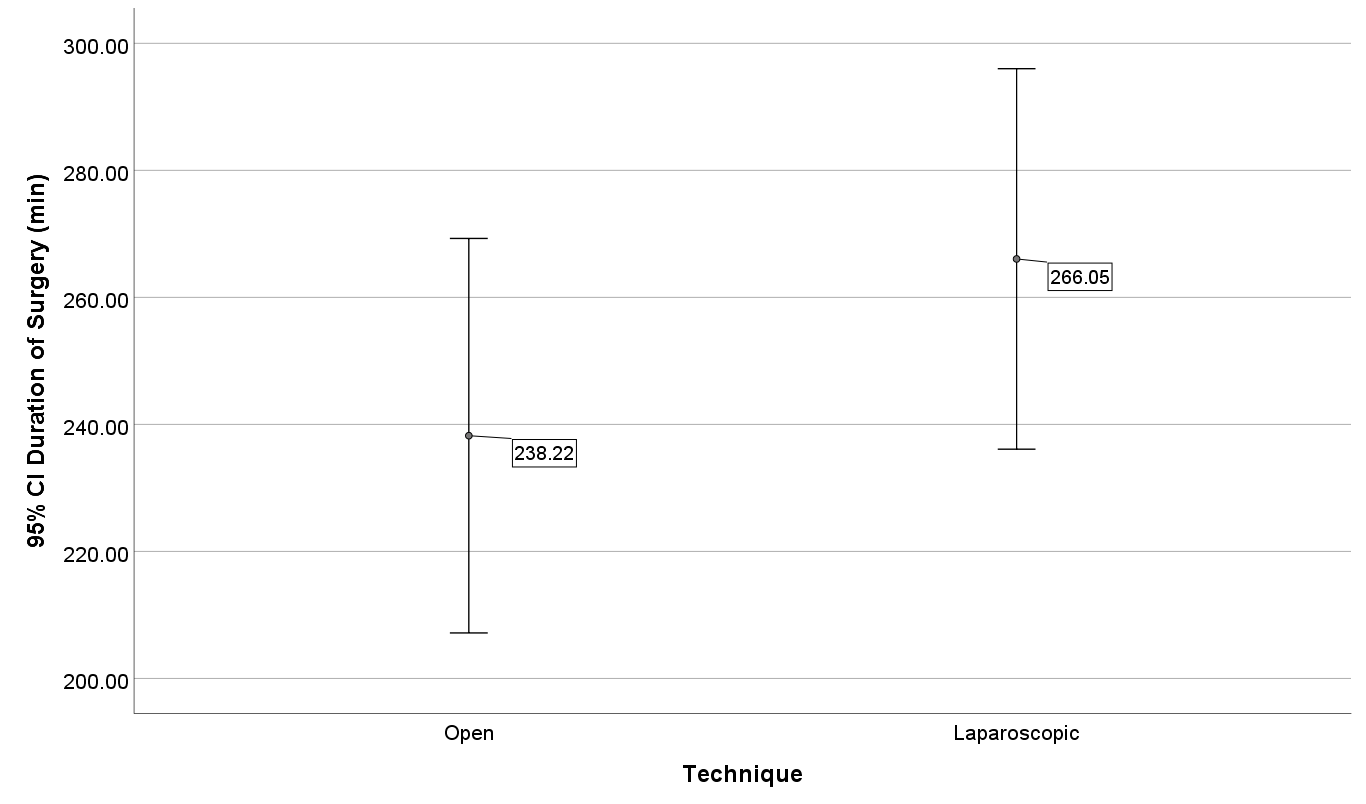


Figure 3: Graph comparing the mean Pringle time (with 95% Cl) between open and laparoscopic major hepatectomies


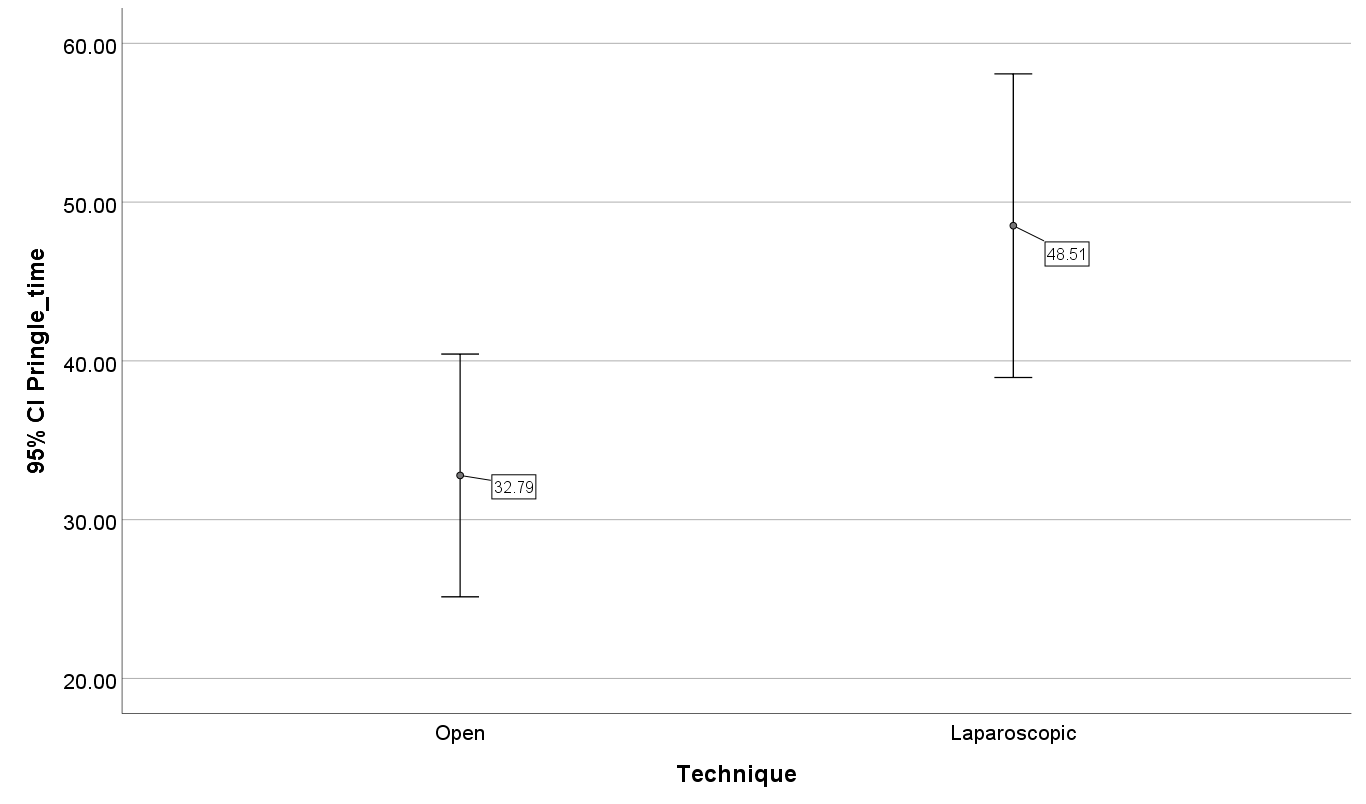


Figure 4: Graph comparing the mean hospital stay (with 95% Cl) between open and laparoscopic major hepatectomies


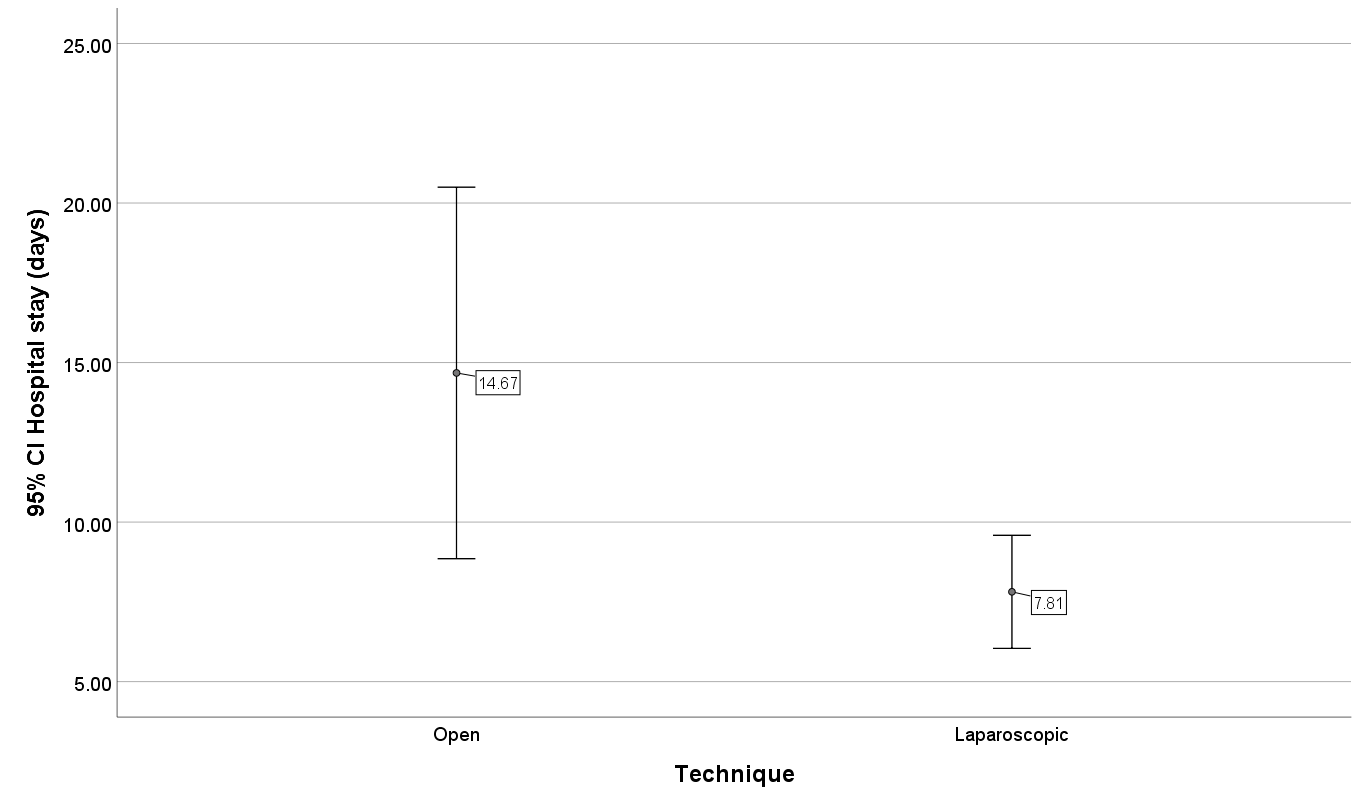

Supplement: Supplementary file 1 [file SupplementaryFile1.docx]
